#  Gαq Signaling Activates β-Catenin-Dependent Gene Transcription

**DOI:** 10.61186/ibj.3890

**Published:** 2023-05-31

**Authors:** Sara Ansari, Sedighe Kolivand, Sara Salmanian, Marie Saghaeian Jazi, S Mahmoud A Najafi

**Affiliations:** Department of Cell and Molecular Biology, School of Biology, College of Sciences, University of Tehran, P.O.Box 14155-6455, Tehran, Iran

**Keywords:** β-catenin, Wnt signaling, G proteins

## Abstract

ABSTRACT

**Background:**

The canonical Wnt signal transduction or the Wnt/β-catenin pathway plays a crucial role in both carcinogenesis and development of animals. Activation of the Gαq class of Gα proteins positively regulates Wnt/β-catenin pathway, and expression of Gαq in HEK293T cells or *Xenopus* oocytes leads to the inhibition of GSK-3β and cellular accumulation of β-catenin. This study investigated whether Gαq-mediated cellular accumulation of β-catenin could affect the transcriptional activity of this protein.

**Methods:**

HEK-293T and HT-29 cells were used for cell culture and transfection. Protein localization and quantification were assessed by using immunofluorescence microscopy, cell fractionation assay, and Western blotting analysis. Gene expression at the transcription level was examined by quantitative reverse transcriptase/real-time PCR method.

**Results:**

Transcription of two cellular β-catenin target genes (*c-MYC *and *CCND1*) and the β-catenin/TCF reporter *luciferase* gene (TopFlash plasmid) significantly increased by Gαq activation. The Gαq-mediated increase in the expression level of the β-catenin-target genes was sensitive to the expression of a minigene encoding a specific Gαq blocking peptide. The results of cell fractionation and Western blotting experiments showed that activation of Gαq signaling increased the intracellular β-catenin protein level, but it blocked its membrane localization.

**Conclusion:**

Our results reveal that the Gαq-dependent cellular accumulation of β-catenin can enhance β-catenin transcriptional activity.

## INTRODUCTION

Specificity in signal transduction pathway is mediated through many known and unknown mechanisms. One of these mechanisms is the cross-talk between the components of two or more signaling pathways. Both the heterotrimeric G protein and canonical Wnt/β-catenin signaling pathways play essential roles in various cellular processes and deregulation of these pathways leads to abnormalities in animal development, as well as many human diseases, including cancers^[^^1^^-^^3^^]^. 

Activation of the canonical Wnt signaling pathway initiates by interacting the Wnt glycoproteins with the Frizzle/LRP5 receptors. While the mechanism of this pathway is still not well understood, its activation results in the inhibition of GSK activity and stabilization of cellular β-catenin protein^[^^1^^,^^2^^]^. Translocation of β-catenin into the nucleus and its interaction with the TCF/LEF transcription factors leads to the transcriptional regulation of the genes involving in diverse cellular processes. In addition, β-catenin has an important role in maintaining epithelial tissues by interacting with the cadherin cell-cell adhesion proteins^[^^4^^-^^5^^]^. 

Given the structural similarity between GPCRs and Frizzled receptors in terms of possessing a heptahelical transmembrane domain^[^^5^^]^, the possibility of regulating Wnt signaling by heterotrimeric G-proteins has been investigated by several researchers who demonstrated that heterotrimeric G-proteins regulate both canonical and non-canonical Wnt signaling pathways^[^^6^^-^^11^^]^. Formerly, we have also shown that activation of the Gαq class of G proteins in two different cell systems (*Xenopus* oocytes and HEK293T cells) leads to the inhibition of GSK activity and accumulation of β-catenin in the cell membrane^[^^12^^,^^13^^]^. 

In this study, we have examined whether the Gαq-mediated increase in cellular β-catenin protein level affects the β-catenin-dependent gene transcription. Consistent with our previous results, we herein showed that Gαq signaling induces the transcription of the β-catenin target genes. Moreover, we provided preliminary results supporting that Gαq signaling increases the intracellular β-catenin level, at least partially, by inhibiting the membrane localization of this protein. 

## MATERIALS AND METHODS

**Cell culture and transfection**

HEK293T and HT-29 cells were respectively grown in DMEM and RPMI 1640 medium supplemented with 10% FBS and antibiotics (100 µg/ml of streptomycin and 100 U/ml of penicillin) in 5% CO_2_ at 37 °C. HEK293T cells are responsive to the canonical Wnt signaling and have therefore been used extensively by the researchers in the field of Wnt signaling pathways. At 60% confluency, the medium of HEK-293T cells was replaced with the fresh medium, and then two hours later, the cells were transfected with the expression plasmids. The standard calcium phosphate protocol was used for transfection^[13]^. Six hours post transfection, the medium was changed, and 48 hours later, the cells were harvested. The cell pellets were used directly or stored at -70 °C until use. Treatment of cells with carbachol (100 µM) or thrombin (0.5 U/ml) was performed 3 and 10 hours before the cell harvest, respectively. 

**Cell fractionation and Western blotting analysis**

Isolation of cytoplasmic and membrane proteins from HEK293T cells, as well as measurement of protein concentration, were performed as described before^[13]^. Briefly, the cells were suspended in a buffer containing 50 mM of Tris-HCl (pH 7.8), 100 mM of NaCl, 2 mM of EDTA, 1 mM of DTT, 0.5 mM of PMSF, 1.5 µM of pepstatin A and lysed by passing them 25 times through a 27-gauge needle. The lysates were centrifuged at maximum speed in a refrigerated microfuge (15.339 ×g) for 15 min, and the supernatants containing the cytoplasmic proteins were transferred to a new tube and stored at -70 °C until use. The pellet containing membrane proteins was rinsed using the above-mentioned buffer and centrifuged, accordingly. The washed pellet was dissolved in 120 µl of 50 mM of Tris-HCl (pH 7.8), 100 mM of NaCl, 1 mM of EDTA, 1 mM of DTT, 2% SDS, 100 mM of β-mecaptoethanol, 10% glycerol, 0.5 mM of PMSF, and 1.5 µM of pepstatin A. The suspension was heated in a 95 °C water bath for 4 min, vortexed and heated again at 95 °C for 3 min. The suspension was centrifuged (15.339 ×g) at RT for 30 min, and the supernatant was preserved as membrane proteins. Protein concentration was measured using a previously described method^[14]^. To this end, 20 µg of proteins from each fraction was separated by SDS-PAGE (8%) and transferred to a nitrocellulose membrane. Following electrical transfer, the gel was stained with Coomassie blue overnight to verify transfer efficiency. The membrane was washed using TBS (25 mM of Tris-HCl [pH 7.5], 137 mM of NaCl, and 2.7 mM of KCl) for 15 min and incubated in fat-free milk 5% (in TBS) at RT for 1 hour to block the nonspecific binding. The blot was then incubated with a polyclonal primary antibody against β-catenin at a 1:1000 dilution at RT for 1 hour. The blot was again washed four times (1, 5, 10, and 15 min, respectively) with TBS and incubated for another one hour with horseradish peroxidase-labelled goat anti-rabbit IgG (1:1000 dilution) at RT. The membrane was finally washed in TBS for 15 min, and the antigen-antibody complex was visualized using an ECL detection kit (Pierce, USA). 

**Indirect immunofluorescence**

Indirect immunofluorescence experiments were performed as described previously^[13]^. In summary, HEK293T cells grown on glass coverslips were washed in PBS, fixed in 4% paraformaldehyde and permeabilized in 0.2% Triton X-100 in PBS for three minutes. The coverslips were washed with PBS and then incubated in blocking buffer (3% BSA) for 30 min before being incubated in a 1:500 dilution of β-catenin antibody at RT for 1 h. The coverslips were again rinsed three times (10 min each) in PBS and then incubated with FITC-conjugated secondary antibody (1:250) at RT for 1 h. The coverslips were finally washed and visualized by a fluorescence microscope (Zeiss, Germany). 

**Quantitative reverse transcriptase and real-time PCR experiments**

 HEK293T cells were harvested 48 hours post transfection with Trypsin/EDTA (0.53 mM of EDTA and 0.05% [w/v] Trypsin in PBS) and washed twice with cold PBS. Total RNA was extracted from the cell pellets using the RNX-Plus kit (CinnaGen, Tehran, Iran) as described by the supplier. Next, 2 µg of RNA was treated with 1 U of DNase I in a total volume of 10 µl at 37 °C for 30 min. The DNase enzyme was inactivated in 2.5 mM of EDTA at 65 °C for 10 min, and then the reaction was used for reverse transcription by adding 200 U of reverse transcriptase enzyme (Fermentase, USA), 1× RT buffer, 20 U of RiboLock RNase Inhibitor, 1 mM of dNTP mix, and 0.2 µg of random hexamer primer in a total volume of 25 µl. PCR amplification was performed on 1 µl of the reverse transcription reaction using 30 pmol of each primer and 2.5 U of Taq polymerase in a total volume of 25 µl. The amplification protocol included denaturating at 95 °C for 60 s, annealing (*GAPDH* and *c-MYC* at 59 °C, *CCND1* at 57 °C, and *luciferase* at 62 °C) for 60 s, and extension at 72 °C for 60 s. The 30 cycles of PCR were followed by a final extension at 72 °C for 10 min. PCR products were separated on a 1% agarose gel and visualized by ethidium bromide. The results of RT-PCR experiments were quantified by the Image J software. Real time-PCR experiments were performed using the QuantiFast™ SYBR Green PCR Master Mix kit (Qiagen, Germany) under the following programs: initial denaturation at 95 °C for 5 min, followed by 40 cycles of denaturation at 95 °C for 15 s, annealing at 60 °C for 25 s, and extension at 72 °C for 25 s. For each 10 µl reaction, 1 µl of complementary DNA (100 ng) was used with 1 µl of each primer (1.0 µM) plus 5 µl of SYBR Green I Master Mix on a real-time thermo cycler Rotor Gene 6000 (Qiagen). Analysis was performed using Corbett rotor-gene 6000 software based on the comparative Ct method. The relative amount of the target gene expression was quantified relative to the reference gene (*GAPDH*) expression. The primers are listed in Table 1.

**Statistical analysis**

All the experiments were repeated at least three times. The data were expressed as means ± SE. Statistical data analysis was performed using SPSS 16.0 and Prism 8 software. Unpaired student t-test and one-way ANOVA were used for comparing the groups. To compare differences in gene expression between the groups, nonparametric Kruskal-Wallis H and Mann-Whitney U tests were applied. p values ≤ 0.05 was considered statistically significant. 

**Table 1:** Oligonucleotide primers used for RT-PCR and real-time PCR experiments

**Gene **	**Primers**
*GAPDH*	F: 5’ CCA GGT GGT CTC CTC TGA CTT CAA CAG 3’ R: 5’ AGG GTC TCT CTC TTC TTC CTC TTG TGC TGC 3’ F: 5’ AAG GTG AAG GTC GGA GTC AAC 3’ R: 5’ GGG GTC ATT GAT GGC AAC AAT A 3’
	
*CCND1*	F: 5’ TTC CTC TCC AAAATG CCA G 3’ R: 5’ AGA GAT GGA AGG GGG AAA GA 3’ F: 5’ GAG GGT TGT GCT ACA GAT GA 3’ R: 5’ CGC CTC CTT TGT GTT AAT GC 3’
	
*c-MYC*	F: 5’ CAC CAA CAG GAA CTA TGA CC 3’ R: 5’ CGC AGA TGA AAC TCT GGT TC 3’ F: 5’ GGC TCC TGG CAA AAG GTC A 3’ R: 5’ AGT TGT GCT GAT GTG TGG AGA 3’
	
*luciferase*	F: 5’ CTC ATA GAA CTG CCT GCG TG 3’ R: 5’ GGC GAA GAA GGA GAA TAG GG 3’

F, forward primer; R, reverse primer. When two pairs of primers were designed for gene amplification, the underlined primers were used specifically for real-time PCR experiments.

## RESULTS

**Activation of Gαq signaling increases the expression of** **β** **-catenin target genes**

To evaluate β-catenin transcriptional activities, we selected two native cellular genes, *CCND1 *(the cyclin D1-encoding gene) and *c-MYC* plus the reporter *luciferase* gene cloned into the TopFlash plasmid^[^^15^^]^.

**Fig. 1:**
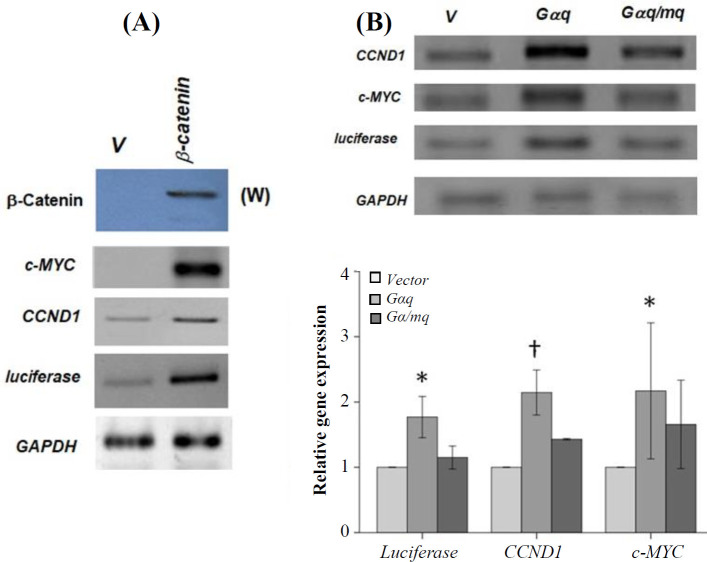
(A) HEK293T cells were transfected with empty vector (V) or the β-catenin-encoding plasmid and used for RT-PCR experiments to amplify the *c-MYC*, *CCND1*, *luciferase*, and *GAPDH* genes. The Topflash plasmid, carrying several β-catenin/TCF target elements, was employed to measure the expression of the reporter *luciferase* gene^[^^14^^]^. The top panel (W) is the result of a Western blot experiment, showing the expression of exogenous β-catenin; (B) Expression of two native β-catenin target genes (*CCND1* and *c-MYC*) and the β-catenin-responsive *luciferase* gene in HEK293T cells transfected with the nonrecombinant (v), *Gαq*, and *mq* plasmids. Fourty eight hours post transfection, RT-PCR assay was performed. *Gαq* and *mq* represent the plasmids encoding Gαq and the Gαq blocking peptide, respectively. The chart shows the average of three independent experiments (^*^ p < 0.05 and ^†^ p < 0.1). The result of one of the RT-PCR gels is shown above the chart. V; nonrecombinant vector

**Fig. 2:**
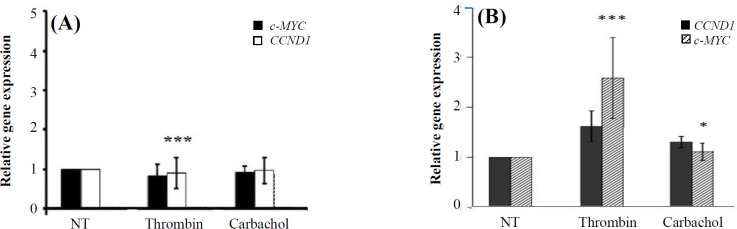
HEK293T (A) or HT-29 (B) cells were treated with 0.5 U/ml of thrombin (10 h) or 100 µM of carbachol (3 h) and used for real time-PCR experiments to measure the expression of the *c-MYC* and *CCND1 *genes. The charts represent an average of three independent experiments (^***^p < 0.001 for thrombin vs. non-treated cells [NT]).

Although it has been shown that the mentioned genes are responsive to the β-catenin/TCF complex^[^^15^^-^^18^^]^, we measured their transcription in the presence and absence of exogenous β-catenin to further verify that the expression of the chosen genes can be induced by β-catenin (Fig. 1A). We have previously shown that the expression of Gαq or activation of the endogenous Gαq increases intracellular β-catenin protein level^[^^12^^,^^13^^]^. Therefore, we initially used quantitative reverse transcriptase PCR to test whether Gαq expression affects the transcription of the β-catenin target genes or not. As shown in Figure 1B, the expression of Gαq increased the transcription of the target genes (*luciferase* [p = 0.014], *c-myc* [p = 0.037], and *CCND1* [p = 0.1]) by about two-fold. This increase was blocked by the expression of a Gαq minigene, which encodes a short peptide corresponding to the C-terminal 11 amino acids of Gαq. The specificity of this peptide in blocking Gαq has previously been verified^[^^13^^,^^19^^,^^20^^]^. We then examined if endogenous Gαq activation could induce β-catenin target gene expression. In this regard, we treated HEK293T cells with thrombin or carbachol. Thrombin is a known agonist for PAR1, a GPCR that preferentially couples to Gq^ [^^21^^]^. Carbachol is a cholinergic agonist that activates M1/3 muscarinic acetyl choline receptors, the GPCRs that also couple to Gq^[^^22^^]^. We have previously shown that treatment of HEK293T cells with these two agonists leads to more than two-fold increase in the cellular protein level of β-catenin^[^^13^^]^. Treatment of HEK293T cells with these agonists (0.5 U/ml thrombin or 100 µM carbachol) did not significantly change the expression of the β-catenin target genes (Fig. 2A). Expression of the reporter *luciferase* gene was also not affected by the treatment of these cells with carbachol (data not shown). Although there are reports that HEK293T cells endogenously express both PAR1 and muscarinic receptors^[^^23^^,^^24^^]^, the expression levels may not be sufficient to induce Gαq-mediated β-catenin transcriptional activity. To verify this assumption, we used HT-29 colon cancer cells, which express higher levels of several Gq-coupled GPCRs including PAR1, as compared to the corresponding normal cells^[^^25^^]^. As shown in Figure 2B, while treatment of HT-29 cells with carbachol slightly increased *CCND1* and *c-MYC* gene transcription, the thrombin treatment led to about 2- to 2.5-fold increase in the expression of these two genes (Fig. 2B).

**Activation of Gαq signaling appears to block** **β** **-catenin cell membrane localization**

**Fig. 3:**
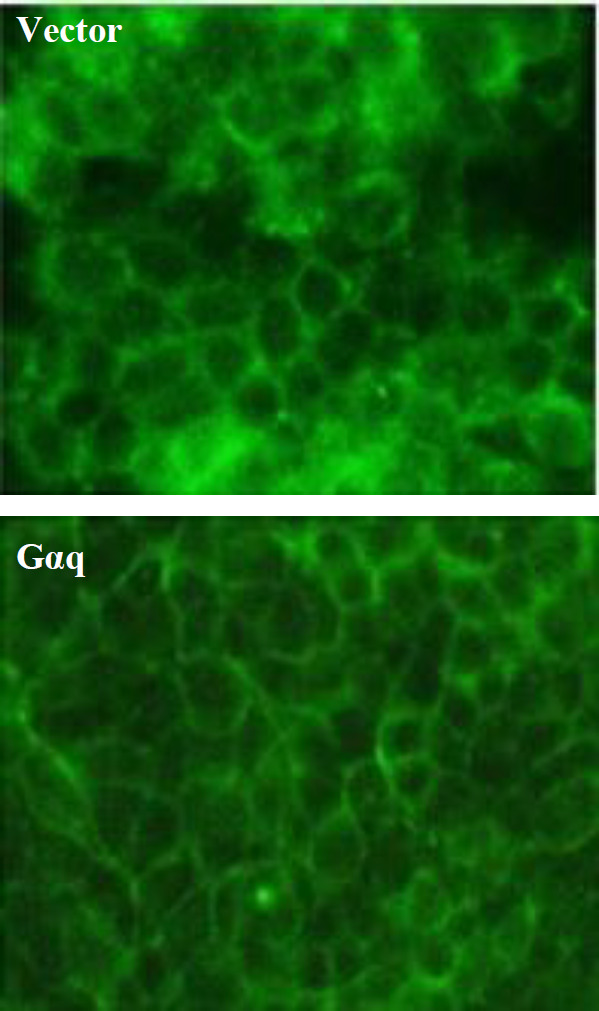
HEK-239T cells transfected with empty vector or Gαq-encoding plasmid and used for immunofluorescence microscopy experiment using β-catenin antibody.

**Fig. 4:**
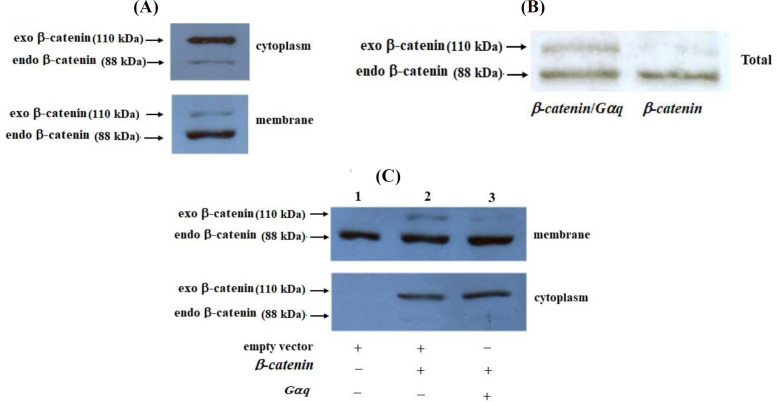
HEK293T cells transfected with a Myc-tagged β-catenin-encoding plasmid (A) with or without the Gαq-encoding plasmid (B). Fourty eight hours post transfection, the cells were collected, fractionated and used for Western blotting experiments. (C) HEK293T cells were transfected with β-catenin-encoding plasmid alone or together with the Gαq-encoding plasmid. Total cellular proteins were extracted 48 h post transfection and used for Western blotting analysis. Exo, exogenous; endo, endogenous

As shown in Figure 3, in HEK293T cells, β-catenin is mainly present in the cell membrane and the expression of Gαq in these cells has no effect on the membrane-localized β-catenin. However, it was interesting to examine the membrane localization of β-catenin when this protein was co-expressed with Gαq. Therefore, we took advantage of an available β-catenin expression construct producing a Myc-tagged version of the protein. The tagged β-catenin protein had a molecular weight of about 110 kDa, 22 kDa larger than that of the wild type native β-catenin (88 kDa) and, therefore, the two forms of the protein could be observed on the blot. The results of cell fractionation and Western blotting experiments showed that when the Myc-tagged β-catenin was expressed in the cells, most of the proteins were localized in the cytoplasm. The protein was also well-localized to the plasma membrane, showing that the 22 kDa Myc tag peptide does not inhibit the membrane localization of β-catenin (Fig. 4A). When Gαq was co-expressed with the Myc-tagged β-catenin, although the total amount of the tagged β-catenin increased in the cell (Fig. 4B), the membrane level of this exogenous β-catenin clearly decreased (Fig. 4C). 

## DISCUSSION

We have previously reported that activation of the Gαq class of Gα proteins inhibits GSK activity, which in turn increases the intracellular level of β-catenin protein^[^^13^^,^^14^^]^. However, it has not before been examined whether the Gαq-mediated increase in the β-catenin protein level affects the β-catenin-mediated gene transcription. Therefore, in this study, we attempted to find the answer to this question. For gene expression experiments, two native cellular β-catenin target genes (*c-MYC* and *CCND1*) plus the reporter *luciferase* gene (under control of the TCF/LEF-binding elements, pTopflash) were selected^[^^15^^]^. As we intended to assess β-catenin transcriptional activity, RT-PCR (quantitative and real-time PCR) experiments were used to measure the *luciferase* gene transcription. Altogether, our results clearly showed that the Gαq signaling activation induced β-catenin transcriptional activity.

In this study, we also found that the Gαq activation could inhibit β-catenin membrane localization (Fig. 4). This is a very interesting preliminary observation, which definitely needs further investigation. Upregulation of β-catenin through Gαq signaling has also been reported by other researchers. In this regard, it has been shown that Frizzled1-mediated differentiation of F9 teratocarcinoma cells (a process that requires β-catenin/TCF-LEF pathway) is dependent on Gαq signaling^[^^7^^]^. Furthermore, it has been reported that treatment of F9 teratocarcinoma cells with Wnt-3a activates a Gαq-mediated phosphatidylinositol signaling, which results in generating inositol polyphosphates such as IP5^[^^10^^]^. Evidence has suggested that transient accumulation of IP5 inhibits GSK-3β and, therefore, leads to the accumulation of β-catenin and TCF/LEF-dependent transcription^[^^10^^]^. Moreover, Gαq signaling is required for the Wnt-mediated disruption of Axin/GSK-3β interaction, which may result in β-catenin cellular stabilization^[^^8^^]^. β-catenin is a known proto-oncoprotein, which its upregulation is involved in tumorigenesis of several human cancers including colorectal cancer. It is believed that this protein is a highly potential target for cancer prevention and therapy^[^^26^^-^^28^^]^. In addition to the Gq class of heterotrimeric G-proteins, the involvement of other classes of G-proteins in the regulation of β-catenin expression and function has been reported^[^^29^^]^. GPCRs are the most diverse class of proteins in mammals involved in many critical cellular processes. Around 900 different GPCRs are encoded by human genome^[^^3^^,^^30^^]^, which are the target of more than 30% of the approved therapeutic drugs^[^^30^^,^^31^^]^. Regulation of β-catenin by heterotrimeric G-proteins raises an interesting question: would it be possible to target G-protein signaling pathways in order to control the expression and function of β-catenin in human cancers? The answer to this question definitely needs much further investigation. Fortunately, the data on the deregulation of G protein signaling pathways in human cancers is growing, and several excellent review articles have already been published, supporting that these important classes of proteins can be considered as potential targets for cancer therapeutics^[^^3^^,^^29^^-^^33^^]^.

In conclusion, the activation of Gαq signaling not only increases the intracellular β-catenin protein level but also results in the regulation of β-catenin nuclear activity. The present study clearly shows that the expression of the wild-type Gαq or activation of the endogenous Gαq causes transcriptional upregulation of two known β-catenin cellular target genes (*c-MYC* and *CCND1*) and a β-catenin-responsive reporter gene. Since β-catenin deregulation is associated with many human cancers, our results further support this hypothesis that trimeric G-proteins and their receptors are among the potential targets for cancer therapy.

## Ethical statement

 Not applicable.

## Data availability

 Data supporting this article are included within the article and supplementary file.

## Author contributions

SA, SK, SS, and MSJ: contributed to experimental design and also performed experiments; SMAN: contributed to experimental design, supervised the project, and wrote the manuscript. 

## Conflict of interest

 None declared.

## Funding/support

This work was supported in part by a grant from Iran National Science Foundation, INSF (93004354) to S Mahmoud A Najafi and also by the University of Tehran Research Department, Tehran, Iran.
